# Case of Supposed Extra-Uterine Fœtation

**Published:** 1829-01

**Authors:** George Jewel


### EXTRA-UTERINE FŒTATION.

### *Case of supposed Extra-uterine Fœtation.*

By George Jewel, Esq.

Mrs. T., residing in the neighbourhood of Manchester-square, a robust healthy woman, has been married two years, and has once aborted. In the month of September, 1827, she quitted her husband, who resides in London, having accepted a situation in the country. At the expiration of a few weeks, she received permission to come to town for a day to see her friends, and, as a natural consequence, passed the night (November 8th) with her husband; the following morning returning to her situation in the country. Soon afterwards, the various sympathetic affections of pregnancy manifested themselves: the morning sickness and heartburn were not only present, but occasioned at times great suffering. The breasts became enlarged and painful, and the areola extended. The catamenia, however, returned at the regular periods, but the secretion was scanty, and of a paler colour than formerly. She now quitted her situation, and returned to her husband, and engaged an intelligent midwife to attend in her approaching confinement.

The circumstance of quickening occurred in the last week of February, and, as is not unfrequent, it was accompanied by a severe paroxysm of fainting. The abdomen gradually enlarged, and the movements of the child, as imagined, could not only be detected with the hand, but were visible to the eye.

On the 9th of August, making a period of 274 days, she was seized with the usual premonitory symptoms of labour: there was pain in the back, a frequent inclination to void the urine, and a slight mucous discharge from the vagina. The midwife was sent for, and soon arrived: she found the patient walking the chamber, and concluded, from her general appearance, and from the presence of all the phenomena of labour, that the process had considerably advanced. Three distinct uterine contractions occurred after the arrival of the midwife, the last of which, from its severity, caused the patient to grasp firmly the back of a chair, and upon its subsiding she became very faint. From that period no further parturient effort was made.

It is necessary to remark, that no examination was made per vaginam; neither was there observed any sanguineous vaginal discharge.

A few days after this occurrence, I first saw the patient, and, having made the usual examination, the os and cervix uteri were not found to have undergone any change. The general health of the patient continues much disordered. The catamenial secretion, still pale, at the last period, did not exceed in quantity a few drachms. The breasts are becoming flaccid, but occasionally painful. She complains of pain in the left hypogastrium, particularly towards night, or when the abdomen is compressed by the hand or by her stays. The abdominal tumor is about the size of a woman's who had arrived at about the seventh month of utero-gestation. She also complains of a weight in the abdomen, particularly upon leaning forwards; and there is a strong impression on her mind that there is "something to come away," or that she might be relieved by an operation.

I am fully aware that females, at the period of life when the catamenia are about to cease, and more particularly if they are very desirous of having a family, often imagine themselves to be pregnant; and that occasionally a variety of anomalous symptoms arise, which are very analogous to the sympathetic affections of pregnancy; but I am not aware that the uterus ever takes on an expulsive action without conception, either uterine or extra-uterine, had actually taken place, or unless it had to get rid of some extraneous body. The nature of the case may be questioned from the circumstance of the uterus not having secreted or expelled its deciduous membrane; but the absence of this tunic would not prove my surmises to be erroneous. Mr. BURNS, a great authority, says that in *most instances* decidua is formed. Dr. BLUNDELL, whose researches have been extensive, examined two cases in which the decidua were wanting. Mr. LANGSTAFF, in an examination of a woman post mortem, in a similar case, could not find any deciduous membrane. The strongest feature of the case is, the patient sleeping one night only with her husband, and the uterus taking on its parturient action precisely at the termination of the nine calendar months.

*Sackville street; Dec. 2d,* 1828.

---

